# Ranking Network of a Captive Rhesus Macaque Society: A Sophisticated Corporative Kingdom

**DOI:** 10.1371/journal.pone.0017817

**Published:** 2011-03-15

**Authors:** Hsieh Fushing, Michael P. McAssey, Brianne Beisner, Brenda McCowan

**Affiliations:** 1 Department of Statistics, University of California Davis, Davis, California, United States of America; 2 Department of Statistics, University of California Davis, Davis, California, United States of America; 3 California National Primate Research Center, University of California Davis, Davis, California, United States of America; 4 California National Primate Research Center, Department of Population Health and Reproduction, School of Veterinary Medicine, University of California Davis, Davis, California, United States of America; University of Maribor, Slovenia

## Abstract

We develop a three-step computing approach to explore a hierarchical ranking network for a society of captive rhesus macaques. The computed network is sufficiently informative to address the question: Is the ranking network for a rhesus macaque society more like a kingdom or a corporation? Our computations are based on a three-step approach. These steps are devised to deal with the tremendous challenges stemming from the transitivity of dominance as a necessary constraint on the ranking relations among all individual macaques, and the very high sampling heterogeneity in the behavioral conflict data. The first step simultaneously infers the ranking potentials among all network members, which requires accommodation of heterogeneous measurement error inherent in behavioral data. Our second step estimates the social rank for all individuals by minimizing the network-wide errors in the ranking potentials. The third step provides a way to compute confidence bounds for selected empirical features in the social ranking. We apply this approach to two sets of conflict data pertaining to two captive societies of adult rhesus macaques. The resultant ranking network for each society is found to be a sophisticated mixture of both a kingdom and a corporation. Also, for validation purposes, we reanalyze conflict data from twenty longhorn sheep and demonstrate that our three-step approach is capable of correctly computing a ranking network by eliminating all ranking error.

## Introduction

In many animal societies, individual members are understood to possess an intrinsic characteristic called *dominance* which both regulates and is molded by their interactions with other members [1]. Dominance hierarchies are thus a common feature of many such societies, ranging from wasps [2] to crayfish [3] to pigeons [4] to longhorn sheep [5], [6]. Furthermore, animal societies have typically been placed into one of two categories: those with defined dominance relationships and those with undefined, or egalitarian, relationships, e.g., [7]. In social groups with detectable dominance relationships, group members are usually arranged into a sequential (often called *linear*) hierarchy based upon observations of direct conflicts between group members. In a sequential hierarchy, the top-ranked member of the group dominates all other members, the second-ranked member dominates all other members except the top-ranked member, and so on. An abundance of mathematical methods have been presented to determine the sequential hierarchy in animal societies based on observed interactions among their members, e.g., [2], [6], [8], [9].

However, in some social groups the dominance hierarchy may not follow a completely sequential pattern, particularly in larger groups and those with highly complex social and cognitive abilities. In addition, the social hierarchy may exist along a continuum between a sequential dominance hierarchy and an egalitarian society where some group members have clear dominance relationships while others do not [2], [9]. Therefore, alternative methods for analyzing the ranking structure of social groups must be sought.

**Figure 1:**
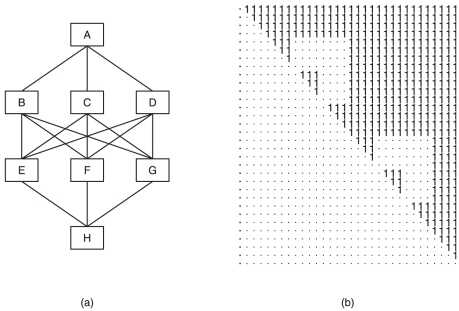
An illustrative matrix representation of an ideal corporative ranking structure. Panel (a) depicts a hierarchy of four tiers with eight groups. Groups on the same tier have the same rank, Panel (b) shows an upper-triangular matrix consisting mostly of 1 s, with several rectangular patches consisting of 0 s (represented by dots), representing the presence or absence of pairwise dominance relationships among the members of the eight groups.

A common example of a non-sequential network is the corporative one. A corporative ranking network allows several groups of individuals to have no dominance relations among them. Typically such a network consists of several tiers: one “governing” group sitting on the top tier, and multiple lower tiers, each of which may contain several parallel groups. Sequential dominance relationships are only found among individuals within the same group and among groups with a group-dominance relation. This ranking network is common in human society, but has rarely been studied using social network analysis [10]. An illustrative network is given in panel (a) of Figure 1. A special case of a corporative ranking network is the kingdom ranking network, in which each group is identified by a common biological linkage, such as matriline or common genetic descent. In particular, the “governing” group is the so-called “royal” group, and royal group members are absolutely not dominated by any non-royal group member in the society.

A distinctive feature of a non-sequential ranking network in general is that transitivity, by which a dominance relationship between two individuals may be inferred based upon their observed interactions with a common third party, plays a more influential role than that in a sequential ranking network. For instance, if 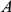 dominates 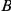, and 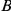 dominates 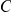, then it is inferred that 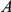 dominates 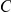. With 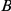 as an intermediate individual, the dominance relationship between 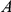 and 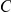 can hold even without observation of direct interactions between 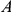 and 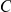. Thus, in a corporative ranking network, many network parameters, such as dominance potentials, are highly interdependent and interact in a non-sequential fashion. The task of constructing such a complex network, therefore, becomes computationally challenging.

The pertinent challenge can be perceived from two successive levels: The first level involves simultaneous inference of all possible dominance potentials among network members from observed behavioral interactions. These behavioral data involve pairs of network members with and without observed direct interaction, and thus the task of estimating the dominance potentials of all possible pairs is subject to highly heterogeneous measurement error. The second level involves identifying each individual's network coordinate, by arranging all pairwise dominance potentials into an upper-triangular matrix form similar to the illustrating example presented in Figure 1(b).

In the matrix shown in Figure 1(b), a 1 in cell 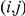 indicates that the 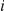
th individual dominates the 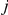
th individual in the society, while a dot indicates otherwise. The rows (and columns) are arranged in such a way that the first four correspond to the members of group 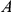 at the top of the structure shown in panel (a) of Figure 1, followed by four rows corresponding to the members of group 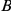, then group 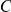, and so on, with the final four rows corresponding to the members of group 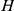. The rectangular patches of dots within the upper triangle occur because the pairwise dominance between members of different groups occupying the same tier of the hierarchy, such as groups 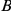, 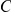 and 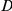, cannot be distinguished. However, within any group the members may follow a dominance order, which is indicated by the consecutive small triangular clusters of 1 s along the diagonal of the matrix. The absence of 1 s in the lower triangle shows that a permutation of the members of this society has been identified such that, if the members are assigned ranks in this order, then no individual of lower rank dominates any individual of higher rank.

Let 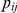 denote the dominance potential of group member 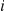 over member 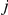, with 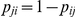. Then 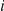 dominates 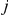 if and only if 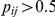. Hence, given a matrix 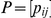 with the rows and columns to be ordered by rank, the goal is to find a permutation of all individuals in the society such that the sum of the dominance potentials exceeding 0.5 in the lower triangle of the matrix are as small as possible. The individual ranks of each network member must be simultaneously estimated based on both direct observation of dominance behavior and inferred dominance due to the assumed transitivity. Thus the challenge becomes a high-dimensional mathematical optimization problem subject to heterogeneous uncertainty. Moreover, since in a non-sequential ranking network many pairs will have dominance potentials near 0.5, more than one set of ranking coordinates can produce a legitimate optimal solution.

The fact that non-sequential ranking network structure has not yet been well-studied in the literature is partly due to the unavailability of reliable data. However, the availability of reliable data has changed dramatically in the past two decades as the awareness of social network theory, aided by information advances, has motivated researchers to collect network-like data, especially in animal behavioral studies. Reliable behavioral data sets of non-human primate societies are available from many institutes across many research fields, such as the California National Primate Research Center (CNPRC).

In this paper we resolve the aforementioned challenges computationally. Three steps are devised. The first step simultaneously estimates all dominance potentials based on both direct pairwise interactions and transitive dominance inferred from interactions with common third parties. Our second step estimates the ranking coordinates of all individuals by minimizing the cumulative error in the inferred dominance potentials across the entire network. The third step derives confidence bounds for desired empirical features of the computed ranking network by accommodating heterogeneous measurement error.

In addition to its ability to accommodate non-sequential ranking structures, our three-step approach provides advantages not found in other existing mathematical approaches. In our first step, we enhance the initial estimate of the dominance potential, which is based only on direct pairwise interactions, by further incorporating information gained through the assumption of dominance transitivity. By converting from the estimated probabilities to odds and adjusting the odds based on interactions with third parties, we are able to accommodate apparent inconsistencies among the data, such as when member 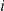 usually wins its conflicts with member 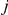 and 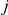 usually wins its conflicts with member 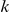, but 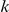 usually wins its conflicts with 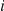 (see [9]). In such cases, the estimated dominance potentials among the three individuals, after converting back from odds, will rightly tend to be closer to 0.5 than they would be had transitivity been neglected. Moreover, in our third step, we take into account the uncertainty in the data in order to provide a measure of precision for our rank estimates. An individual that consistently wins in many conflicts with individual 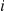 and consistently loses in many conflicts with member 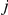 should almost certainly be assigned a rank between the ranks of 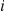 and 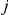. But for many individuals there are few observed conflicts with the other members, and the outcomes of those few conflicts may vary from one occasion to the next. In such cases the ranking coordinates are less certain. This uncertainty is quantified under our approach using repeated samples from a posterior probability distribution, so that confidence bounds for the ranks of individuals may be established. We are unaware of any other method that accounts for the uncertainty in this manner.

In an attempt to validate our proposed three-step approach and at the same time make a comparison with a result based on Bayesian analysis [6], we reanalyze a data set of twenty longhorn sheep from [5]. We show our steps are capable of computing a set of “perfect” non-sequential ranking networks, in which our estimate of 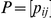 contains no values in the lower triangle exceeding 0.5. The existence of such a set is primarily due to the fact that there are two sheep that have no direct interactions with most of the dominant sheep, but have a couple of wins over lower-ranking sheep. Therefore they can be placed at several different ranking network coordinates without affecting the non-sequential ranking network. This perspective of a ranking network is nearly impossible for a purely sequential ranking model based on Bayesian analysis, even for a relatively small society.

We apply our three-step approach to sets of conflict data, collected at CNPRC, by first establishing a ranking network for 94 adult rhesus macaques (mean total group size = 137 adults, juveniles, and infants) living in a half-acre outdoor captive enclosure. The resultant ranking network is coupled with known matriline information. We then apply our approach to conflict data collected from a second colony of macaques to compute the ranking network and confirm the utility of this methodology. In this fashion we address the question: Is the rhesus macaque's society organized as a purely corporative ranking network, a kingdom version of a corporative network, or a mixture of these? The characterization of rhesus macaque society in terms of corporative or kingdom ranking networks may also shed light on how such hierarchical networks are maintained through lower-level interactions among individuals and groups of individuals (such as matrilines).

## Methods

### Ethics statement

All research reported in this manuscript adhered to the recommendations in the Guide for the Care and Use of Laboratory Animals of the National Institutes of Health, the laws of the United States government, and the recommendations of the Weatherall report, “The use of non-human primates in research.” All research subjects were housed in large social groups in half-acre outdoor enclosures to provide for their psychological well-being. The methodological approach was purely observational, and involved no experimental or invasive treatment of the animals. All occurrences of illness or injury among study subjects were immediately reported to and treated by CNPRC veterinary staff, and all efforts were made to ameliorate suffering. This project was approved by the University of California, Davis Institutional Animal Care and Use Committee, protocol #11843.

Consider a single enclosure with 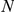 adult rhesus macaques indexed by 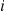 from 1 through 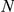. For any 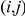 pair of macaques, we want to infer the unknown probability 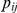 that the 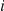
th macaque dominates the 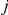
th macaque, as well as the reverse dominance probability 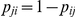. The inference for 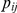 and 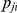 is based on the behavioral records 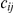 and 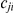, which count the numbers of observed wins for the 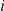
th macaque over the 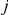
th macaque, and vice versa, among their total number 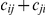 of observed direct conflicts.

Due to the weekly behavioral sampling scheme (6 hrs/day, 4 days/week; see [11], in press, for a complete description of observational methods), it is not uncommon to find severe heterogeneity in the collection of data 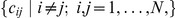. In a cage consisting of 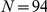 mature macaques, the total possible number of pairwise interactions within the social group is 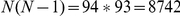. But only a small percentage of pairs were observed to have any direct interaction, and the remaining pairs were never observed to interact. Hence, under modeling with Bernoulli random variables, the unknown parameters 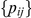 would be estimated with very high heterogeneous precision for those cases of 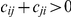. However, their estimated dominance probabilities may differ significantly from 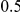, because of the transitivity property. Thus, the collection of 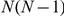 parameters are dependent upon one another in a non-sequential fashion, and not orthogonally, as is often assumed. The parameter space, where the “true” parameters live, is indeed a very complex manifold.

Given the non-sequential interdependence among the collection of parameters in such a network, the likelihood-based approaches, including maximum likelihood and Bayesian analysis, are inappropriate for proper inference on the 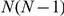 parameters, simply because the optimization task upon a nearly 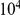-dimensional complex manifold is practically impossible. A glimpse of such difficulty can be seen in the Bayesian analysis reported in [6]. Even when ignoring the interdependence among the parameters, the high dimensionality alone, despite the small group size of 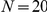, causes the posterior density to be too flat to be of practical use.

The first task in computing a non-sequential ranking network is to simultaneously calculate the 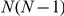 dominance potential parameters for all possible pairwise interactions among network members. Since many of these pairs have no observed direct interactions, the dominance potential parameters must be inferred from the transitivity relationships among the parameters. These features create very high dimensionality in the parameter space and result in high heterogeneity of precision in parameter estimation. We address this task in the first subsection.

After estimating the 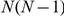 parameters, the next challenging task is to construct a non-sequential ranking network. This task is analogous to working out a puzzle with many irregularly-shaped pieces and many missing pieces. We accomplish this in the second subsection by transforming the network construction task into an optimization problem, and then developing an approach equipped with Simulated Annealing [12] to resolve this fundamental construction. Finally, we present a third step in the third subsection in which we derive a basis for empirical confidence bounds for features observed in the computed ranking network.

### The first step: S1

Given the 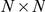 matrix 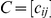 of behavior records for each pair of macaques, we construct four new 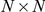 matrices. Matrix 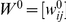, with 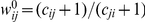 for 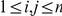, gives the empirical odds that macaque 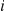 dominates macaque 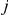, based on observed confrontations between the two. We add one to each record to avoid division by zero. Then matrix 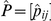, where 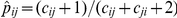, gives the empirical probability that macaque 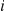 dominates macaque 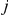 based solely on direct observations of conflicts. The computed 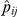 serve as initial estimates of the unknown probabilities 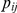, which are the true pairwise dominance probabilities.

Here 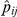 may be regarded as the posterior mean of a beta distribution with parameters 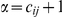 and 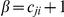. That is, given a uniform prior distribution for the unknown probability 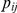 and a binomial distribution for 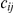 (with parameters 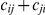 and 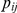), the posterior distribution of 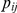 given 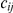 is the Beta 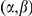 with mean 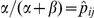. This idea will be exploited in step S3.

From 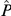 we construct the transitivity structure matrix 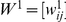 such that
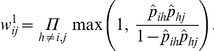
Here 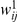 signifies the dominance odds between macaques 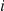 and 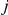 based solely on transitivity implied by their interactions with other single intermediate macaques. Note that 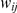 is never less than one. In order for the ratio 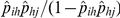 to exceed one, the observed dominances of macaque 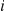 over intermediate macaque 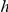 and of macaque 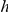 over macaque 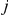 must both be strong enough to make the product 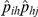 exceed 0.5. Hence the dominance of macaque 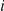 over macaque 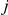 via transitivity is implied conservatively. The matrix 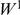 thus carries information about the dependence network structure embedded in the data.

Finally, we construct the matrix 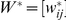 by first setting 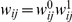 for 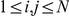, then setting 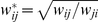 to standardize. This standardization makes 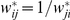. The matrix 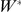 thus gives the odds that macaque 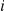 dominates macaque 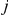, implied by both observation of direct conflicts between them and by the transitivity relations among the other macaques. Step S1 outputs both 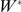 and 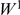 for use in the next two steps.

### The second step: S2

The second step estimates the rank of each macaque in the social hierarchy based on the dominance odds computed in step S1. This is accomplished by first computing an initial ranking using the matrix 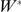 obtained in S1, then using the simulated annealing algorithm to improve upon this ranking by minimizing a cost function.

Based on the 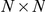 matrix 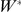 from S1, with elements 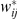, we construct the matrix 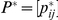, where 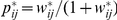 for 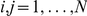. This converts the odds into enhanced probabilities. These probabilities 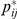 differ from the probabilities 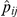 computed directly from the observed pairwise conflicts, since the dominance probability implied by transitivity has been incorporated into their computations. We then choose a threshold 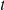 and construct the matrix 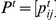 such that 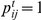 if 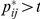 and equals zero otherwise. In practice we use 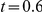. We seek to rearrange the columns of 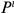 such that the upper triangle contains few zeros and the lower triangle contains many zeros. To accomplish this, we compute the sum of each column, then order the columns such that the sums of the columns are nondecreasing from left to right. We then rearrange the rows and columns of 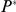 according to the same order. The purpose of this effort is to minimize the sum of those values in the lower triangle of 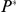 exceeding 0.5. The values in the lower triangle represent the estimated probabilities that lower-ranking macaques dominate higher-ranking macaques, which would not exceed 0.5 in the ideal situation. This technique produces the initial estimate of the macaque ranking order, which is not necessarily optimal.

**Figure 2:**
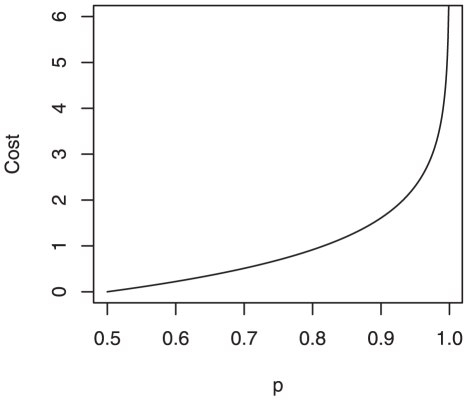
Plot of cost function for 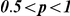.

To determine the quality of the estimated ranking order, we devise a cost function whose argument is the lower diagonal entries of the matrix 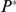. We construct this function so that values greater than 0.5 in the lower diagonal will be penalized, with the penalty increasing at a greater rate as the values approach one. While other definitions are plausible, the cost function used here is defined as
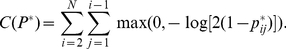
(1)As Figure 2 shows, for each element 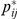 in the lower triangle of 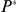, this function adds cost equal to 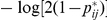 whenever 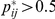, with the cost increasing steeply as 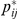 approaches one. In the ideal situation, no element in the lower triangle of 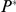 exceeds 0.5, and thus the cost is zero. But observations of conflict behavior in a complex society may not allow the computation of an optimal cost near zero. The simulated annealing (SA) algorithm [12] is implemented to seek an optimal ranking order that will provide a total cost which is as close to zero as possible.

In the SA algorithm, we start at an initial ranking order. We then consider a “neighbor” ranking order, in which the ranks of two randomly selected macaques are switched. If the cost function decreases when 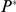 is modified by rearranging the rows and columns according to the neighbor ranking order, we move to the new ranking order. Otherwise, we move to the new ranking order with probability 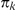. We compute 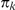 based on our progress in the SA algorithm, so that if we plan to complete 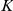 iterations, 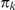 will decrease exponentially from nearly 1 to almost 0 as 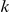 approaches 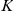. In practice we use 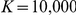 and decrease 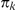 only when 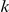 is a multiple of 10.

After the last iteration we save the ranking order which produced the lowest cost encountered during the SA algorithm. The SA algorithm is designed so that it will tend to find the global minimum on a surface that may have multiple local minima. We run the SA algorithm many times and choose the best overall ranking order, i.e., that which corresponds to the rearrangement of rows and columns of 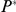 which yields the lowest cost. This order is taken as our estimate of the ranking of the mature rhesus macaques in a given enclosure.

### The third step: S3

The third step is used to construct a basis for empirical confidence intervals for desired features of the estimated ranking order obtained from step S2. For all 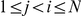 we randomly draw 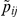 from the beta distribution with parameters 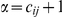 and 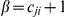. For 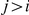 we set 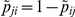. Note that the mean of the given beta distribution is 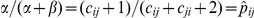. Hence 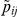 represents a random perturbation of the empirical dominance probability 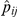 of macaque 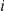 over macaque 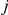. Once we obtain the matrix 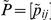 we set 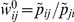 to convert the probability into odds, and thus construct the matrix 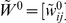. Using the transitivity structure matrix 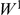 constructed in step S1, which carries the transitivity structure that characterizes the real data, we construct the matrix 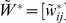 by first setting 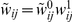 for 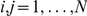, then setting 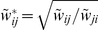 to standardize. We run step S2 on 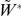 to obtain an optimal ranking order 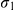. We then repeat with another draw from the beta distribution to obtain 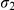, and so on to obtain 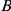 optimal ranking orders 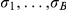.

We may then examine the distribution of the estimated rank for any individual macaque among the 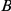 ranking orders to derive confidence bounds for the rank estimate obtained in step S2. We can also derive bounds for the range of rankings corresponding to a specified matriline within the macaque society, or for other features of interest.

## Results

### Female longhorn sheep

A data set consisting of pairwise wins and losses within a group of 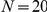 female longhorn sheep originally from [5] was analyzed in [6] using Bayesian analysis under the sequential ranking network framework. The matrix in Table 1, reproduced from [6], shows in entry 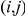 the number of times sheep 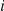 defeated sheep 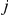 in confrontations. This matrix, arranged in ranking order, possesses several typical features of observational studies: few interactions for most pairs, and many pairs with no interactions. By assuming the existence of a sequential ranking hierarchy among the 20 female sheep, Bayesian analysis is conducted to estimate the twenty individual dominance potentials, say 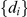, 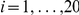. The estimation is based on the likelihood constructed under an independence assumption among all pairs as
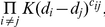
where the logistic version of the Bradley–Terry model [13] sets 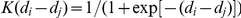 as the kernel for transforming the difference between dominance potentials into a winning probability. The twenty-dimensional, mutually-independent normal flat priors of 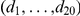 are used. An estimate of 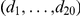 is permuted and gives rise to a sequential ranking hierarchy.

**Table 1:** Conflict data for female bighorn sheep.

ID	15	17	21	06	08	01	10	22	02	11	05	16	12	09	26	25	29	27	28	24
15	0	0	4	0	7	0	0	1	1	0	3	0	4	1	1	2	2	9	2	5
17	0	0	3	0	1	0	0	0	0	1	2	0	5	1	0	0	0	2	1	1
21	0	0	0	0	2	0	1	1	1	1	1	1	1	1	3	0	1	3	2	1
06	0	0	0	0	0	0	0	1	0	0	0	0	3	0	1	0	0	1	1	1
08	0	0	0	1	0	1	0	2	3	2	1	0	1	1	2	2	2	6	4	0
01	0	0	0	0	0	0	0	0	0	0	0	0	0	0	1	0	1	0	0	0
10	0	0	0	0	0	0	0	0	0	0	2	0	0	0	0	0	0	1	0	0
22	0	0	0	0	0	0	0	0	2	1	2	0	1	0	2	0	0	1	2	3
02	0	0	0	0	0	0	0	0	0	0	1	2	1	4	1	0	0	1	0	0
11	0	0	0	0	1	0	0	0	0	0	0	0	1	0	1	0	0	1	0	1
05	0	0	0	0	0	0	0	0	0	0	0	0	0	0	0	1	1	2	1	1
16	0	0	0	0	0	0	0	0	0	0	0	0	0	0	0	0	0	0	1	0
12	0	0	0	0	0	0	0	0	0	0	5	0	0	0	0	1	1	1	0	0
09	0	0	0	0	0	0	0	0	0	0	0	0	0	0	0	1	0	0	1	0
26	0	0	0	0	0	0	0	0	0	0	0	0	0	0	0	1	1	0	1	0
25	0	0	0	0	0	0	0	0	0	0	0	0	0	0	0	0	0	0	1	0
29	0	0	0	0	0	0	0	0	0	0	0	0	0	0	0	0	0	0	1	0
27	0	0	0	0	0	0	0	0	0	0	0	0	0	0	0	0	0	0	0	1
28	0	0	0	0	0	0	0	0	0	0	0	0	0	0	0	0	0	0	0	3
24	0	0	0	0	0	0	0	0	0	0	0	0	0	0	0	0	0	0	0	0

Matrix of wins and losses for 20 female bighorn sheep, *Ovis canadensis*, with rows and columns arranged according to estimated dominance rankings [5], [6].

Unfortunately, the posterior density of 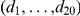 from this Bayesian analysis is just too flat to give rise to a meaningful sequential ranking hierarchy. In contrast, our three-step approach computes several zero-cost permutations among the 20 female sheep. The most obvious one is (15, 17, 21, 08, 06, 01, 10, 22, 02, 11, 16, 12, 05, 09, 26, 25, 29, 27, 28, 24), which pushes down the locations of sheep numbers 06 and 05 in the Adams result. Indeed, when the data are displayed according to this permutation, all but one entry in the lower-triangle of the matrix of Table 1 are zeros. When the estimated dominance probability matrix 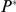 is arranged in this order, no values in its lower triangle exceed 0.5, so that its cost is zero. Hence this computational result implies that the above permutation produces a perfect ranking order, while the Bayesian result is flawed.

Typically a macaque conflict data set, as we will analyze in the next section, does not allow for such perfection when it comes to ranking. Usually there are many seemingly contradictory wins and losses that further complicate the task of ranking, particularly in species with frequent alliances and/or bidirectional aggression (see, e.g., [14]). However, such complexity is well-expected in a more sophisticated society. Hence it is deduced here that Bayesian analysis and the sequential ranking assumption are not suitable for a data set embedded with such kinds of complexity, especially when 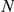 is as large as 100.

### Ranking network for rhesus macaques

**Figure 3:**
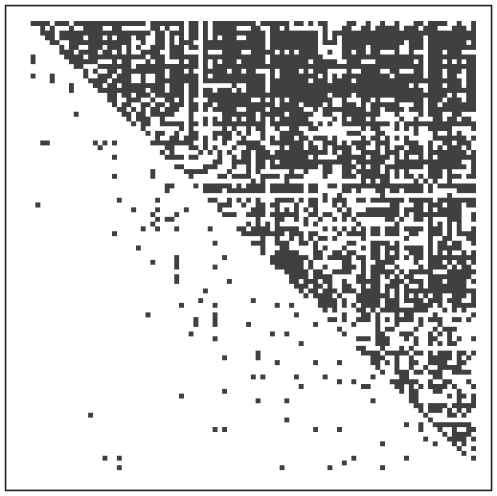
Computed rhesus macaques' dominance probability matrix, with rows and columns arranged based on the optimal ranking order, for Cage 5. A black square indicates a computed dominance probability above 0.6.

We apply our three steps to conflict data collected on two outdoor captive groups of rhesus macaques housed at the CNPRC. These behavioral data were collected between June 2008 and April 2009, and include all aggressive interactions that had a decisive outcome (for detailed methods, see [11]). For example, an interaction where an initiator threatens a recipient and the recipient runs away is counted as a win for the initiator and a loss for the recipient. Other aggressive behaviors include lunging, chasing, and biting. We apply steps S1 through S3 to these data. The first group (Cage 5) includes 94 adult rhesus macaques. Figure 3 is a graphical representation of the optimal dominance probability matrix 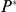 returned by S2. A black square indicates a matrix entry whose value exceeds the threshold of 0.6, which we take to be a strong indicator of dominance. The cost of the corresponding optimal ranking network, using relation (1), is computed to be 55.02. It is noted that the cost for the initial ranking order used in step S2 was 205.62. Thus we see that the Simulated Annealing algorithm achieves a very significant improvement. From the ranking perspective, this network reveals a clear non-sequential structure. Although it does not look exactly like the matrix shown in Figure 1(b), there are many areas in the upper triangle of Figure 3 that are rather sparse. Those sparse areas above the diagonal are likely to indicate parallel groups, while sparse triangular areas along the diagonal are rank-blurring groups. The latter groups' memberships are very essential for this non-sequential ranking network because they constitute the network's backbone structure.

Based upon the optimal ranking network, we list in Table 2 the rank coordinates computed for the members of each matriline in the macaque enclosure (Cage 5). Males are distinguished by bold italics. The most significant finding from this analysis is that the ranking network for this macaque society seems to have a “governing” group consisting of a dominant matriline (C7) subordinate only to a lone alpha male, and several additional elite individuals of either gender from several lower-ranking matrilines. This “power-sharing” structure seems to be very similar to human political systems. This sophisticated structure may be one of the key factors underlying the group's stability. Another significant finding, based on the sparse sections visible along the middle of the diagonal of Figure 3, is the existence of a subgroup of middle-ranking individuals who are somewhat parallel in dominance potential. These sparse sections correspond to the macaques between the 23rd and 50th ranks. Interestingly, this subgroup consists of a single middle-ranking matriline (F10), together with several individuals from other middle-ranking matrilines (D10, S16, J6, L4), as may be concluded from Table 2. All these facts reveal the evident non-sequential structure in the ranking of the 94 individuals.

**Table 2:** Cage 5 rankings by matriline.

Matriline	Ranking coordinates
NRM	***1***
C7	3 4 ***5*** 6 7 8 9 10 12 14 ***15***
X1	11 16 17 20 24 69 ***89 92 94***
S16	18 ***19*** 21 22 23 ***35 42***
D10	26 ***34***
J6	25 29 31 36
L4	30 33 ***38***
F10	27 28 ***37*** 39 ***40*** 41 43 44 45 46 47 ***48 49*** 50 51 52 ***84***
N4	32 ***53*** 54 55 56 57 58 59 60 ***61*** 63 ***65*** 68
M10	***2*** 62 64 ***66 76 77***
Z2	***13*** 67 70 71 72 73 74 78 ***79 80*** 81 82 83 85 86
G8	***75*** 87 88 90 91 93

Summary of ranking coordinates for rhesus macaques in Cage 5 grouped according to matrilines. Males are indicated by bold italics.

In terms of the corporate structure depicted in Figure 1(a), it appears that matriline C7 would correspond with group A at the top level, while matrilines X1, S16 and J6 would correspond with groups B, C and D on the second tier. Matrilines L10, D4 and F10 would share the third tier, corresponding to groups E, F and G. Matrilines N4, M10 and Z2 would comprise a fourth tier that has no counterpart in Figure 1(a), while matriline G8 occupies the bottom level, corresponding to group H.

**Figure 4:**
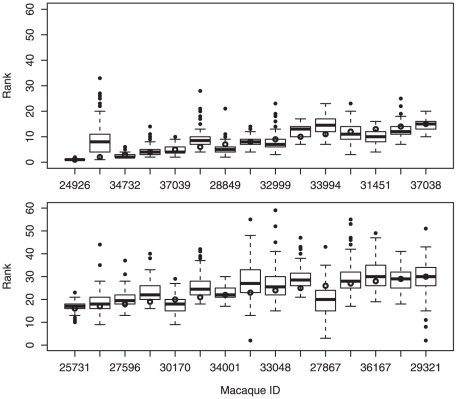
Boxplots indicating confidence bounds for top twenty rhesus macaques, based on optimal ranking order computed in step S2 and 105 iterations of step S3 for macaques in Cage 5.

Based on our output from step S3, which involved 105 iterations, we may infer confidence bounds for the ranking of each macaque. Figure 4 displays boxplots for the top 20 macaques based on the optimal ranking determined by step S2 (indicated by open circles in the figure). Each boxplot indicates the distribution of computed rankings for each of these macaques over the 105 iterations. Points above or below the whiskers of a boxplot are outliers. A short box suggests that the corresponding macaque's rank is fairly consistent over the 105 iterations, as is the case with the alpha male (ID 24926). Hence the rank of such individuals is fairly certain. A long box suggests that the corresponding macaque's estimated rank is quite uncertain, given the data, as with the beta male (ID 22898). Moving this macaque's position in the ranking order does not affect the cost significantly. Since there are few such individuals in this example, the variability of their rankings over the 105 iterations has only a slight impact on the variability of the rankings for their neighbors in the hierarchy.

We might also take interest in the range of ranks for a group of macaques, such as for a particular matriline. Our step S2 estimated the ranks for the eleven macaques in the C7 matriline to fall between 3 and 15 inclusive, making this the highest-ranking matriline in the society. Using the output of step S3, we find that the highest rank assigned among the members of C7 was distributed as follows: 1 (one time), 2 (88 times) and 3 (16 times). Meanwhile, the lowest rank among the C7 macaques was distributed more widely:
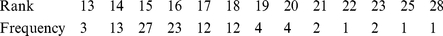
The range between the highest and lowest ranks was centered at 14. To obtain empirical 95% confidence bounds, we eliminate the 5 most extreme values from each distribution, and conclude with 95% confidence that the highest rank for matriline C7 is between 2 and 3, while its lowest rank is between 13 and 22. This gives strong evidence that this matriline dominates the others.

We implement the same procedure for a second macaque colony living in a separate enclosure (Cage 8) at the CNPRC. We initially identify 136 adults in the colony. However, only 91 of these adults were involved in observed conflicts during the period of interest, so the other 45 adults were removed from consideration. The value of the cost function (1) for the optimal ranking network for these macaques, based on the output of step S2, was 49.59. Once again, a lone male is identified at the top of the hierarchy, followed by a dominant matriline (C16). The remaining matrilines follow in succession, with several matrilines having an elite member of either gender whose rank is very high compared to the rest of the matriline. The ranks are arranged by matriline in Table 3, with males indicated by bold italics. In terms of Figure 1(a), we do not find quite the same multi-tiered structure. It is likely matrilines G11, Q3 and D13 occupy the same tier, as do matrilines N8 and N6, while the other matrilines occupy tiers by themselves. Since the analysis of the second colony identifies a similar network structure, our confidence in the consistency of this three-step approach is strongly validated. Our analysis of several additional colonies yield comparable results.

**Table 3:** Cage 8 rankings by matriline.

Matriline	Ranking coordinates
S22	***1***
C16	2 3 6 7 8 9 10 ***13*** 14
F13	4 11 15 16 17 18 19 20 21 22 23 ***43 87***
V5	24 25 26 27 28 29 30 31 34 35 ***90***
L8	36 37 38
K8	12 39 40 41 42 44 45 46 47 49 52 69 ***86 91***
D13	***5*** 48 54
G11	51 55 58 ***59*** 60 61 64 ***89***
Q3	50 53 56 57 63 85 ***88***
T9	65 66
B12	67 68 70 71 72 ***81***
M14	32 73
N8	***62*** 74 75 77 78 ***79***
N6	***33*** 76 80
P11	82 83 84

Summary of ranking coordinates for rhesus macaques in Cage 8 grouped according to matrilines. Males are indicated by bold italics.

## Discussion

We propose a three-step approach to compute and construct a non-sequential ranking network based on highly heterogeneous counts of aggressive interactions among mature members of a society of rhesus macaques. We provide theoretical arguments and explicit reasoning that describe the challenging nature of this task. We propose a set of three steps, each of which is designed to resolve a significant challenge encountered in the construction of real-world ranking networks. However, our solution to this computational challenge sheds light on how the underlying corporative kingdom structure of rhesus macaque society influences the behavior of individuals as well as the stability of the society as a whole. We expect that this new approach will be useful in many other studies, especially when dealing with the property of transitivity among binary relational data. From this perspective, corporative network theory may still be in its infancy.

Finally, we briefly comment on the limits of the maximum likelihood estimate (MLE) approach and Bayesian analysis in constructing corporative ranking networks. The transitivity-induced parameter dependence makes the pertinent parameter space a complex manifold. This manifold structure is problematic for the optimization procedures of the MLE and the assignment of reliable prior distributions in Bayesian analysis. In particular, Bayesian analysis always produces a very flat posterior density when employing a non-informative prior on even a modest number of parameters. In addition, the high dimensionality of this manifold structure further complicates the situation, if not rendering it impossible to resolve. For example, it is common practice among animal behaviorists to impose a sequential ranking assumption on the structure of animal societies [6]. However, the imposition of a sequential rank order ignores the aforementioned difficulties. Indeed, an assumption of a sequential structure implies transitivity, which further necessitates dependence among the parameters, resulting in such difficulties. Therefore, it is our hope that the three steps developed here will be broadly applicable to the study of ranking network structures of many societies.

This three-step approach is an advancement in the challenging task of computing rank coordinates in societies which do not possess a sequential hierarchy. By incorporating information gained through dominance transitivity, we accommodate apparently contradictory cycles in the data to obtain reasonable estimates of pairwise dominance probabilities. We also acknowledge the uncertainty in the data and exploit it so as to provide reasonable confidence bounds for the ranks of individuals. While other mathematical methods assume a sequential ranking order for all members of an animal society, our approach provides more flexibility, in that the computed result allows for some variability in the estimated ranking coordinates. This is because the assigned ranks for some individuals may be interchanged without affecting the value of the cost function.

For societies like the rhesus macaques, this flexibility in our approach is essential. The scientists who observe the macaque societies at the CNPRC are in agreement that their hierarchies are not sequential. The relative ranks of matrilines can be identified by observation, but the ranks of individuals within the matrilines are more difficult to identify, partly because the groups are very large, and partly because some matrilines are genetically fragmented. Hence, while the imposition of an assumption that the hierarchy is sequential is necessary under other accepted ranking methods, it is artificial, and the output from such methods generally conflicts with the observations of researchers. A method based on a corporative kingdom model, as presented here, is coherent with these observations, and is therefore a more realistic tool for the analysis of rhesus macaque ranking networks.
